# Spatial agreement between Goldmann visual field defects and fundus autofluorescence in patients with birdshot chorioretinopathy

**DOI:** 10.1186/s12348-016-0085-0

**Published:** 2016-05-31

**Authors:** Loren S. Jack, Aniruddha Agarwal, Yasir Jamal Sepah, Quan Dong Nguyen

**Affiliations:** Ocular Imaging Research and Reading Center, 985540 Nebraska Medical Center, Omaha, 68198-5540 NE USA

**Keywords:** Birdshot chorioretinopathy, Goldmann visual field, Autofluorescence, Uveitis, Ultra-wide field imaging

## Abstract

**Background:**

The purpose of this paper is to study the spatial agreement between visual field defects and ultra-wide field (UWF) fundus autofluorescence (FAF) in patients with birdshot chorioretinopathy (BSCR). The study is a retrospective, cross-sectional analysis of a university uveitis practice. Eight (8) eyes of five (5) patients with BSCR were included. Inclusion criteria were ability to fixate reliably. Goldmann visual fields (GVF) and UWF FAF were obtained, digitalized, and standardized. Analysis was performed by measuring areas of overlap of hypo-autofluorescent FAF lesions and GVF scotomas within the central 60°. Overlap was calculated as a percentage of the total area of FAF and GVF, respectively. Average areas were also calculated.

**Results:**

The mean age of the subjects was 51 ± 12.28 years (range 38–69 years). 14 ± 23 % of the total lesion area identified as hypo-autofluorescent on FAF overlapped with scotoma. 28 ± 41 % of the GVF scotomas overlapped with hypo-autofluorescent FAF lesions. Average area of FAF hypo-autofluorescence was much larger (15.19 disc areas) than GVF (3.45 disc areas).

**Conclusions:**

There appear to be larger total areas of hypo-autofluorescence on FAF than scotoma evidenced by GVF and only a small amount of overlap. The finding suggests that GVF is relatively insensitive to anatomic loss, which can be detected using FAF. Further studies are required to assess whether this finding holds true for automated white-on-white perimetry. In addition, more selective psychophysical stimuli may have higher sensitivity in detecting early functional loss that accompanies anatomic damage.

## Background

Birdshot chorioretinopathy (BSCR) is a form of inflammatory posterior uveitis characterized by typical clinical and imaging characteristics. This condition is associated with bilateral hypo-pigmented chorioretinal “birdshot” lesions affecting the peripapillary and posterior region [1–3]. Excellent visualization of these lesions is possible using ultra-wide field (UWF) imaging techniques of fundus photography and fundus autofluorescence (FAF) compared to conventional imaging [4, 5]. However, the lack of correspondence between the various imaging techniques has been well recognized in BSCR. Therefore, the clinicians often rely on a number of imaging and diagnostic tests in order to characterize the disease entity [3, 6].

BSCR appears to have a chronic, progressive course with most patients developing functional loss over a long-term follow-up [7]. Visual acuity appears to be a poor indicator of disease activity, necessitating ancillary tests such as electrophysiology and visual field testing [7–9]. Goldmann visual field (GVF) testing is a more widely available tool for evaluation of visual function compared to electroretinogram (ERG), which may be more expensive and time-consuming [10]. While a number of ophthalmology practices have moved from GVF to automated visual field testing for longitudinal follow-up of patients with BSCR, GVF remains an important testing modality [11, 12]. Previous reports suggest that serial, quantitative GVF testing in patients with BSCR may allow detection of change in retinal function following therapeutic interventions [10].

Evaluation of spatial agreement between UWF FAF and GVF has been performed in other retinal pathologies, such as retinitis pigmentosa [13]. However, the relationship between visual function changes on GVF (i.e., scotomas defined by the V4e isopter, as well as other changes such as generalized constriction of the visual field) and autofluorescence patterns on UWF FAF in BSCR has not been established. In the index study, we analyzed the agreement between the hypo-autofluorescent areas on UWF FAF and GVF scotomas in patients with BSCR.

## Methods

The study was conducted at the retina and uveitis services of the Stanley M. Truhlsen Eye Institute, University of Nebraska Medical Center. Institutional Review Board (IRB) clearance was obtained and the study adhered to the tenets of the Declaration of Helsinki. All the study procedures were compliant as per the HIPAA act of 1996. This retrospective, observational case series included patients diagnosed with BSCR from January 2013 to December 2014. The diagnosis was established based on clinical findings, slit-lamp biomicroscopy, and indirect ophthalmoscopic visualization of characteristic yellow-orange ovoid chorioretinal lesions with mild vitritis, as per the International Consensus Conference on BSCR [14]. Best-corrected visual acuity (BCVA) was obtained. Serologic testing of infectious etiologies and human leucocyte antigen (HLA)-A29 testing was performed as indicated. Demographic details and treatment regimens for each patient was noted.

### Imaging protocol

All the patients underwent imaging studies including UWF color fundus photography, fluorescein angiography (FA), and FAF (Optos P200Tx, Optos Inc., Scotland, UK). Sixteen eyes of 8 patients were screened for inclusion in the study. The exclusion criteria were unreliable GVF testing, poor central fixation due to central visual loss and media opacity precluding adequate imaging. GVF testing was performed using Haag-Streit system, Bern, Switzerland, using V4e, I4e, I2e, and I1e test targets. A single, trained operator tested the visual fields to minimize the variability of the tests. Scotoma was defined using the V4e isopter.

### Overlap analysis

The GVF test results were scanned and digitalized using Adobe Photoshop Elements 11.0 (Adobe Inc., San Jose, CA, USA). ImageJ software (National Institutes of Health, NIH, Bethesda, MD, USA) [15] was used to quantify the area of each defect on the digital GVF images in the central 60°. Hypo-autofluorescent areas on the UWF FAF and the scotomas on the GVF were measured in terms of disc diameters using ImageJ software. The area of overlap was determined by aligning the point of central fixation of the GVF with the center of the fovea on FAF and superimposing the physiologic blind spot with the optic nerve. The FAF images were vertically flipped so that these images could be superimposed to the digitalized GVF printouts. The area consisting of the physiologic blind spot on GVF and area of the optic nerve on FAF were excluded from the analysis in order to selectively study pathologic changes detected on imaging. The overlap analysis was performed by two independent graders.

### Statistical analysis

The data analysis was performed using GraphPad Prism 6 (GraphPad Inc., La Jolla, CA, USA). The area of scotomas on V4e isopter was calculated in terms of disc areas on both GVF and FAF. The percentage area of hypo-autofluorescence on FAF that overlapped with the scotomas on GVF was calculated. Similarly, the percentage area of the scotoma that overlapped with the FAF was calculated. Bland-Altman analysis was used to evaluate the agreement between the two techniques in detecting the areas involved by the disease process. Inter-observer agreement for the measured values was calculated using the intraclass correlation coefficient (ICC) test. A *p* value of <0.5 was considered to be statistically significant.

## Results

Data was collected from a total of 16 eyes from 8 patients. Eight eyes from 5 patients were excluded due to ungradable image quality, lack of patient cooperation, poor central fixation, and/or unreliable GVF testing. Eight (8) eyes of 5 patients (2 males) met the inclusion criteria and their clinical data and images were included in the analysis. All the patients included in the study were positive for HLA-A29. The mean age of all 8 subjects was 51 ± 12.28 years (range 38–69 years). The mean best-corrected visual acuity for the included eyes (converted to LogMAR units) measured 0.65 ± 0.39 units. The baseline demographic details of all the patients are listed in Table 1.

**Table 1:** Baseline characteristics of the study population

Patient number	Gender	Age (years)	HLA-A29 positivity	Eye	Best-corrected visual acuity	Duration of disease (years)
1	Female	53	+	OS	20/50	4
2	Male	83	+	OD	20/50	5
				OS	20/50	5
3	Female	63	+	OD	20/400	10
4	Female	67	+	OD	20/200	11
				OS	20/200	11
5	Male	62	+	OD	20/60	18
				OS	20/30	18

**Fig. 1:**
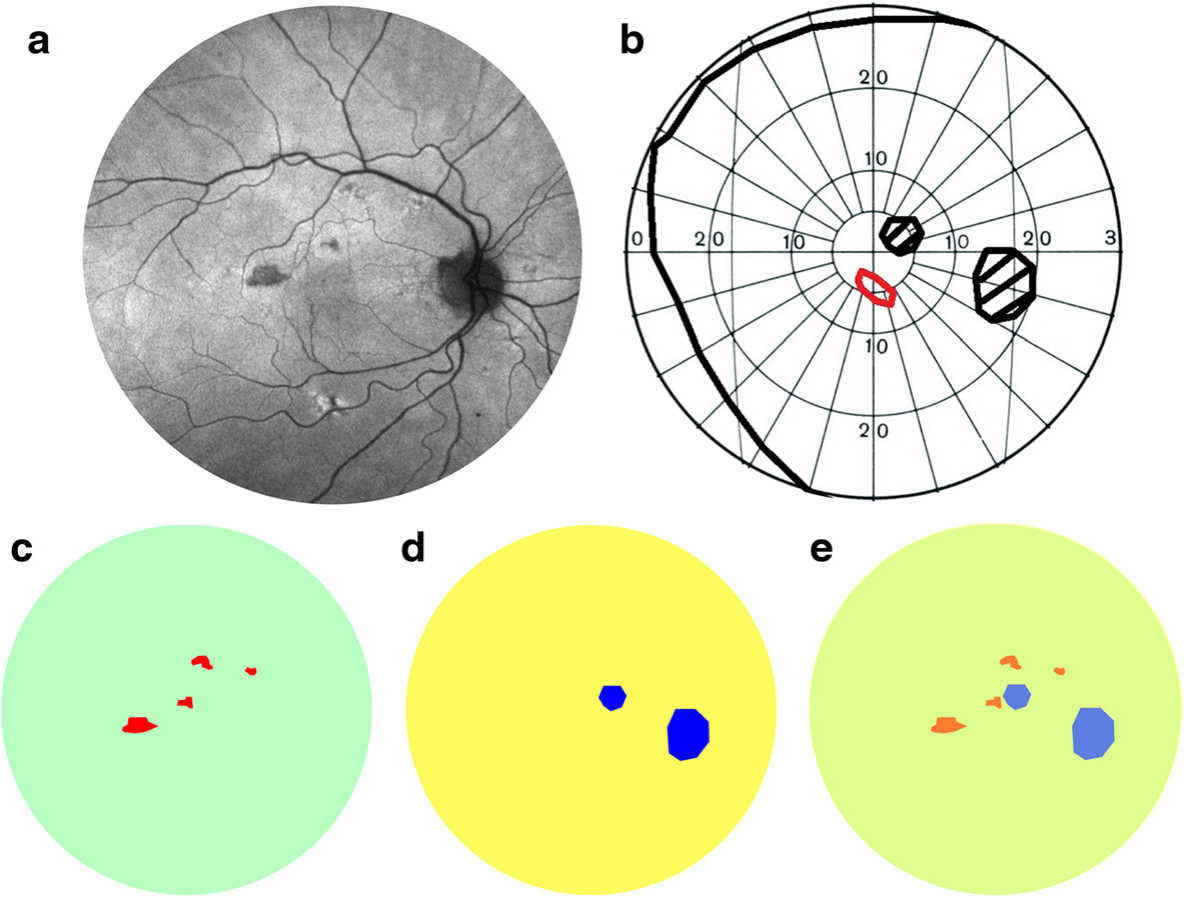
An example of the overlap analysis performed in the study. **a** Fundus autofluorescence (FAF) of the right eye demonstrating hypo-autofluorescent lesions (vertically flipped). **b** Digitized copy of the Goldmann visual field (GVF) cropped to central 60° demonstrating paracentral scotoma. **c** Area of hypo-autofluorescence extracted from the FAF using the red channel. **d** Area of scotoma extracted from the digitized GVF using the blue channel. **e** Overlay of **c** and **d** showing no overlap between the red and the blue channels

**Fig. 2:**
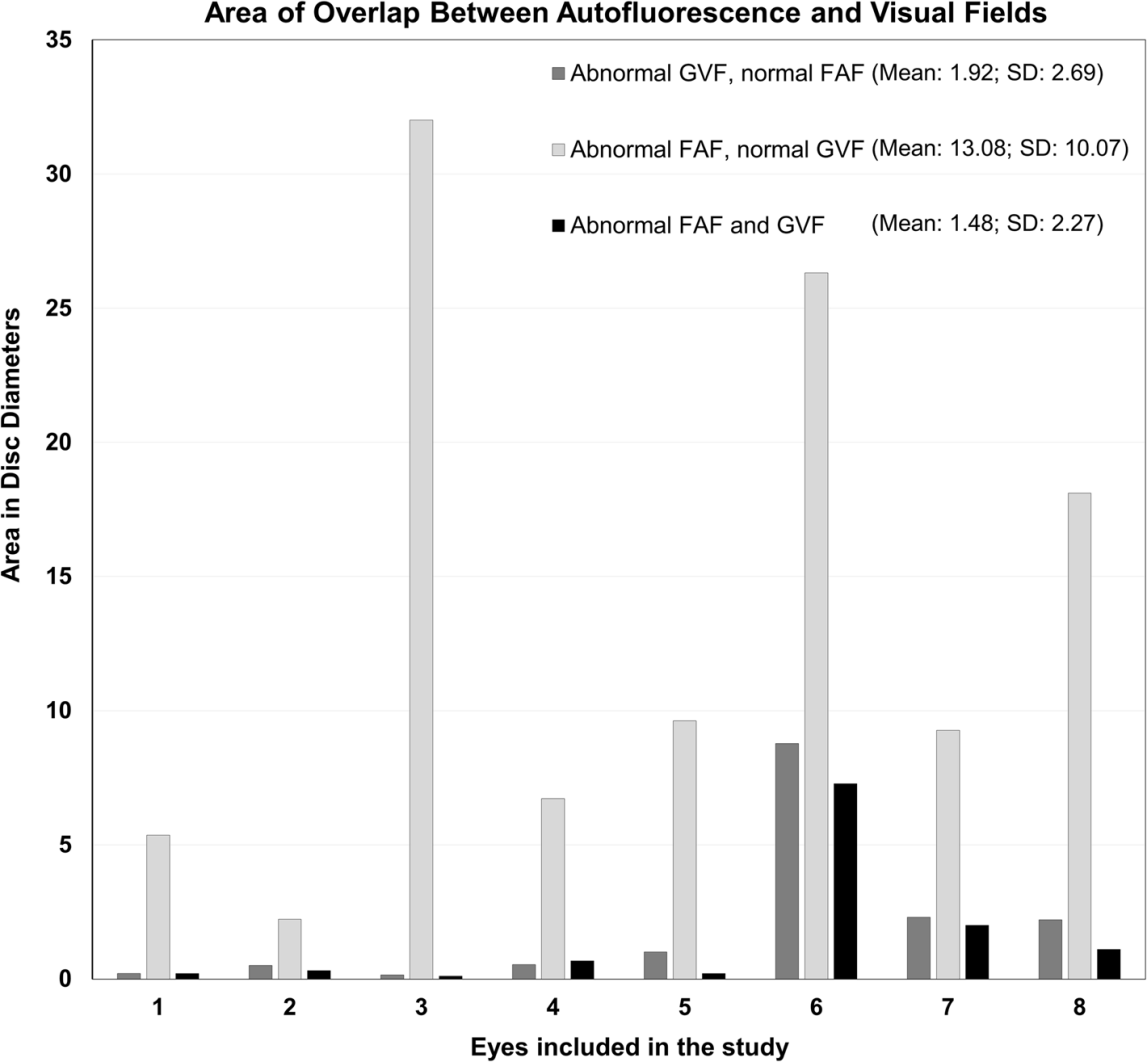
Bar graphs for each eye included in the study showing the area (in disc diameters) of (*1*) abnormal Goldmann visual field (GVF) and normal fundus autofluorescence (FAF), (*2*) abnormal FAF and normal GVF, and (*3*) abnormal GVF and FAF (areas of overlap). The graph shows that there is a relatively small area of overlap between abnormal GVF and FAF and greater extent of FAF loss

**Fig. 3:**
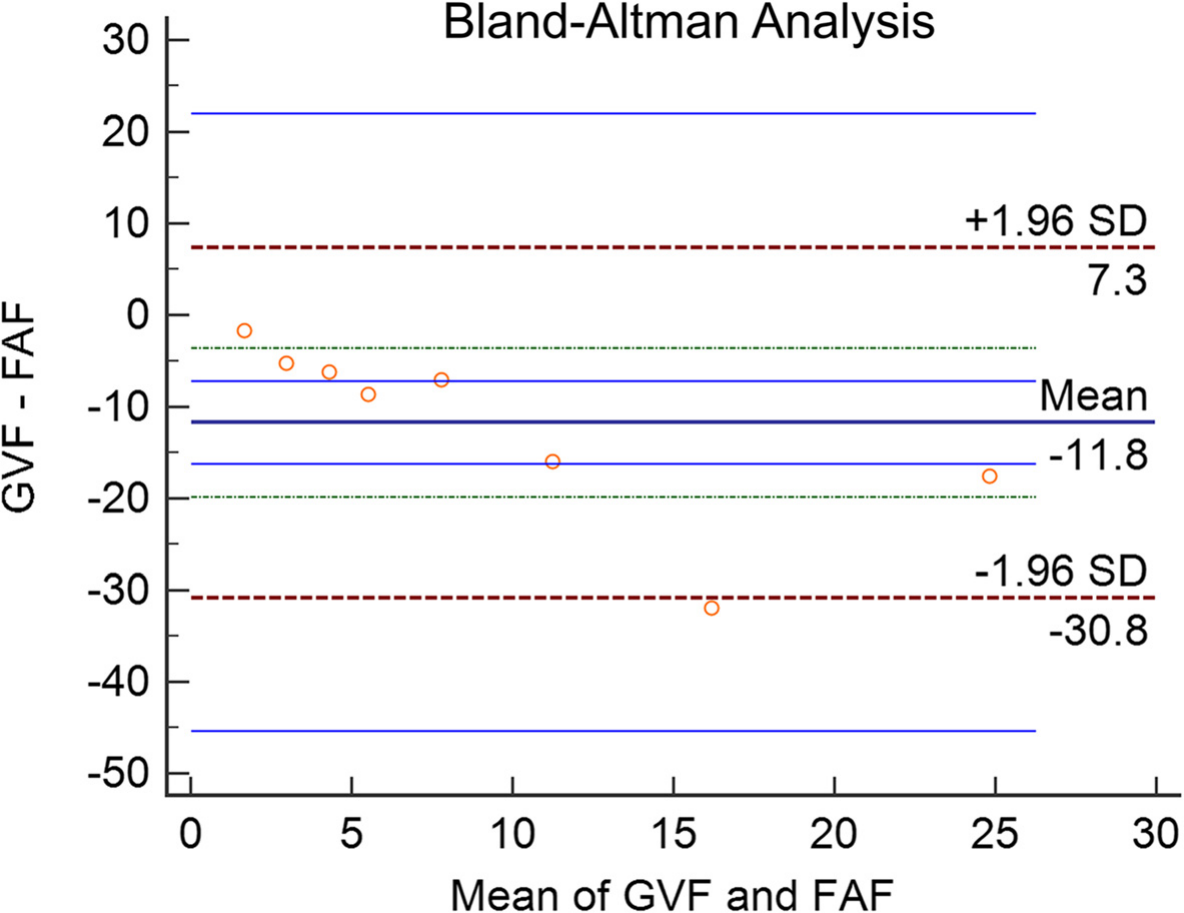
Bland-Altman plot representing the study eyes (*red circles*) showing the mean area of Goldman visual field (GVF) and fundus autofluorescence (FAF) versus the difference (GVF-FAF). The plot demonstrates the individual variation in agreement. The mean difference between GVF and FAF is negative, indicating a greater extent of the area of FAF defects compared to scotomas on GVF

Of the area covered by hypo-autofluorescent lesions identified on UWF FAF, 14 % (±23 %) overlapped with scotoma on GVF. On the other hand, 28 % (±41 %) of the area of GVF scotomas overlapped with the hypo-autofluorescent lesions seen on UWF FAF. An example of the overlap analysis is shown in Fig. 1. Average area of UWF FAF hypo-autofluorescence was 15.19 disc areas and 3.45 disc areas on GVF. Figure 2 is a graphic representation of the overlapping areas between FAF and GVF. The ICC for measurement of hypo-autofluorescent areas on FAF was 0.998; for measurement of GVF was 0.996; and for measurement of the area of overlap was 0.977. The Bland-Altman analysis for the agreement between the two techniques is shown in Fig. 3. The Bland-Altman plot demonstrates the poor agreement between FAF and GVF. In addition, it demonstrates a negative trend: greater mean of GVF and FAF were associated with a more negative GVF minus FAF. Thus, in eyes with more advanced disease, area of hypofluorescence on FAF may exceed GVF loss by a significantly greater extent (Spearman’s Rho = −0.952 for GVF-FAF versus mean GVF and FAF; *p* = 0.0002).

Two distinct patterns of distribution of the hypo-autofluorescent lesions were noted on FAF imaging. The majority of eyes (5 out of 8; 62.5 %) demonstrated macular and extra-macular areas of hypo-autofluorescence. Placoid hypo-autofluorescence involving the macula was noted in one eye. The remaining eyes showed peripapillary areas of hypo-autofluorescence. Unlike the FAF, no consistent pattern of visual field defects was noted on GVF.

## Discussion

BSCR is an uncommon form of posterior uveitis with protean clinical manifestations, often leading to difficulty in diagnosis [3, 16]. The lesions of BSCR may mimic other white dot syndromes, making ancillary imaging tests crucial for the diagnosis and follow-up of patients [17]. Techniques such as spectral-domain optical coherence tomography [18, 19], FA [6], electrophysiology [20], and indocyanine green angiography [21] provide valuable information regarding the structural and functional damage in eyes with BSCR. Wide spectrum of patterns noted on FAF in patients with BSCR helps in the identification of retinal pigment epithelium (RPE) atrophy. Lesions of BSCR may be better appreciated on FAF as compared to clinical fundus photography [17, 22, 23]. Placoid hypo-autofluorescence at the macula in eyes with BSCR has been shown to correlate significantly with poor visual outcome [22].

UWF FAF imaging using Optos scanning laser ophthalmoscopy system provides a non-invasive technique to obtain panoramic, high-quality images in a short span of time [4, 24]. The Optos system uses a wavelength of 532 nm for excitation and detects autofluorescence at 540–800 nm. FAF images obtained using UWF system may provide important information in the assessment and follow-up of patients with BSCR as a wider field of view is obtained [5, 24]. The hypo-autofluorescence on FAF correlates well with the duration of the disease and the degree of inflammation in the affected eye [5].

Visual field loss is common among patients with BSCR. In a previous study by Thorne et al., 75 % of patients had abnormal field scores for the I4 isopter on GVF [10]. Quantitative improvement in vision-related function has been documented using GVF in BSCR following systemic immunosuppressive therapy. In these studies, modified diabetic retinopathy study (DRS) visual field scores were obtained using GVF by calculating the sum of degrees seen along each 30° meridian using I4e and IV41 objects. Using the above method of calculation, a gain of >20° in the visual field was found to occur with immunomodulatory therapy in patients with BSCR [10, 25]. Thus, GVF testing appears to be an efficient objective measure for assessment of vision-related functional loss in patients with BSCR. However, while GVF has been used extensively to study the functional deficit among patients with BSCR, it is important to note that in the present day, automated perimetry may be more commonly employed in the management of BSCR compared to GVF, which may be more tedious, more challenging, and time-consuming to perform repetitively [11, 12].

In the index study, the typical patterns noted on FAF included peripapillary hypo-autofluorescence and scattered macular and extra-macular areas of hypo-autofluorescence, which is consistent with what is reported in literature [4, 5, 15, 21]. Comparison of this structural loss of the photoreceptor/RPE complex using FAF with functional loss using GVF may provide valuable insights into the disease pathophysiology and progression. Using overlap analysis, there appears to be larger total areas of hypo-autofluorescence on FAF than scotoma evident by GVF (15.19 versus 3.45 disc areas). In addition, less than a third of the abnormal area identified by FAF and GVF overlapped. There was a greater percentage of overlap of GVF compared to FAF (28 versus 14 %, despite compensating for the larger areas of hypo-autofluorescence on FAF imaging).

One of the reasons for the differences in the findings of FAF and GVF may be because changes in FAF may *precede* functional loss detected by GVF. Thus, detection of hypo-autofluorescence on FAF may help the clinician in predicting the areas of the retina that may develop loss of function in the future. Such concept is consistent with the observation that significant structural loss at the level of photoreceptors/RPE may be required before functional loss can occur. Our study results may also be partially explained from those observed in glaucoma. Although BSCR and glaucoma are different diseases with very dissimilar pathophysiological mechanisms, the process of visual loss may share certain similarities. In both the conditions, there is slowly progressive visual field loss and involvement of retinal photoreceptors, retinal nerve fiber layer, and the ganglion cells. In studies performed among glaucoma subjects, GVF testing has been shown to be insensitive to early visual field loss with 75 % subjects showing evidence of visual field defects on automated perimetry 1 year before appearing on manual GVF testing [26]. Similarly, Quigley et al. demonstrated that structural damage involving ganglion cells precedes changes on both manual GVF as well as automated perimetry but more so on GVF testing [27]. Thus, from the studies that have been reported, it can be inferred that GVF appears to be an insensitive tool to detect early structural loss. In addition, psychophysical evidence from glaucoma studies have shown that size III white supra-threshold target of white-on-white perimetry (used in both GVF and automated perimetry, including the index study) is insensitive to early field loss in glaucoma. Other testing strategies such as blue-on-yellow perimetry (short-wavelength automated perimetry), frequency doubling technology (FDT), and motion detection techniques have shown to be superior to white-on-white perimetry for early visual loss detection [28]. Similar findings may occur among patients with BSCR, since ganglion cell death among patients with early BSCR may be non-selective just like in glaucoma. Thus, as per the hypothesis of reduced redundancy [28], sparsely represented ganglion cell sub-populations have lower degrees of overlap between adjacent receptive fields than more abundant sub-populations, and may demonstrate identifiable functional loss earlier. According to such hypothesis, we would encourage other clinician scientists to investigate BSCR patients with selective tests of visual function in order to confirm or refute the above hypothesis.

Parallels can be drawn from glaucoma studies to further explain the poor structure-function (FAF-GVF) correlation observed in our study. Evidence suggests that comparing the available imaging and diagnostic techniques may be associated with a number of confounding factors that may limit our understanding of structure-function correlation [29]. Test-retest variability in visual field testing may be a major source of imprecision in determining the structure-function relationship. There may be anatomical factors related to the technique of FAF that may lead to a poor agreement with GVF. Studies have shown that the sensitivity on perimetry may be linearly related to the ganglion cell density in the local area of the retina being tested when both are expressed in linear units [30]. However, hypo-autofluorescence on FAF is related to loss of RPE rather than ganglion cells and thus may not have a linear relationship with perimetric sensitivity, since the RPE consists of non-neural components which are additionally affected in BSCR.

Another reason for the poor correlation between FAF and GVF may be related to the natural history of BSCR. Literature suggests that there may be a lack of correspondence among various imaging tests in eyes with BSCR [3, 17, 18]. This can be explained by the variable involvement of structures such as the choroid, RPE, and the photoreceptors by the disease process as the lesions of BSCR evolve. If the retinochoroidal damage is detected early using techniques such as FAF, it may be possible to halt the progression of the disease with appropriate or escalated systemic immunomodulatory therapy.

Limitations of this study include the retrospective nature of analysis and small sample size. Due to the requirement for fixation, the present study only included patients with the ability to maintain fixation and may not correlate well with patients who have advanced disease and can no longer fixate. The requirement for fixation may also be a source of inclusion bias as patients with higher grades of GVF loss were excluded from the study. Approximately half the eyes were excluded from the analysis due to ungradable image quality or poor patient cooperation/fixation. Such exclusion may represent a source of bias and may affect the overall generalizability of the results. An important aspect to consider is the scale of measurement on GVF and FAF techniques. For example, a loss of 3 dB on GVF represents 50 % loss of sensitivity, since it is a logarithmic scale. On the other hand, FAF has been considered as an all-or-none scale. The difference in scale may represent a limitation in our ability to accurately assess the structure-function relationship [29]. Moreover, our study lacks a normal age-matched control group that may help in determining the specificity of FAF grading since this grading was categorical (normal or abnormal). Additional, prospective studies, including a larger number of patients, are warranted.

## Conclusions

The results of this pilot study underscores the need to follow patients with BSCR using multi-modal imaging as each technique may contribute different information about the disease status. It is relevant to combine structural and functional testing to enhance our understanding of the disease process and relationship between visual loss and underlying tissue damage in diseases such as BSCR.
